# Supplementary figures and images for: The microbiota promotes social behavior by modulating microglial remodeling of forebrain neurons
Source: PLoS Biol. 2022 Nov 1;20(11):e3001838. doi: 10.1371/journal.pbio.3001838 (PMC9624426; doi:10.1371/journal.pbio.3001838)

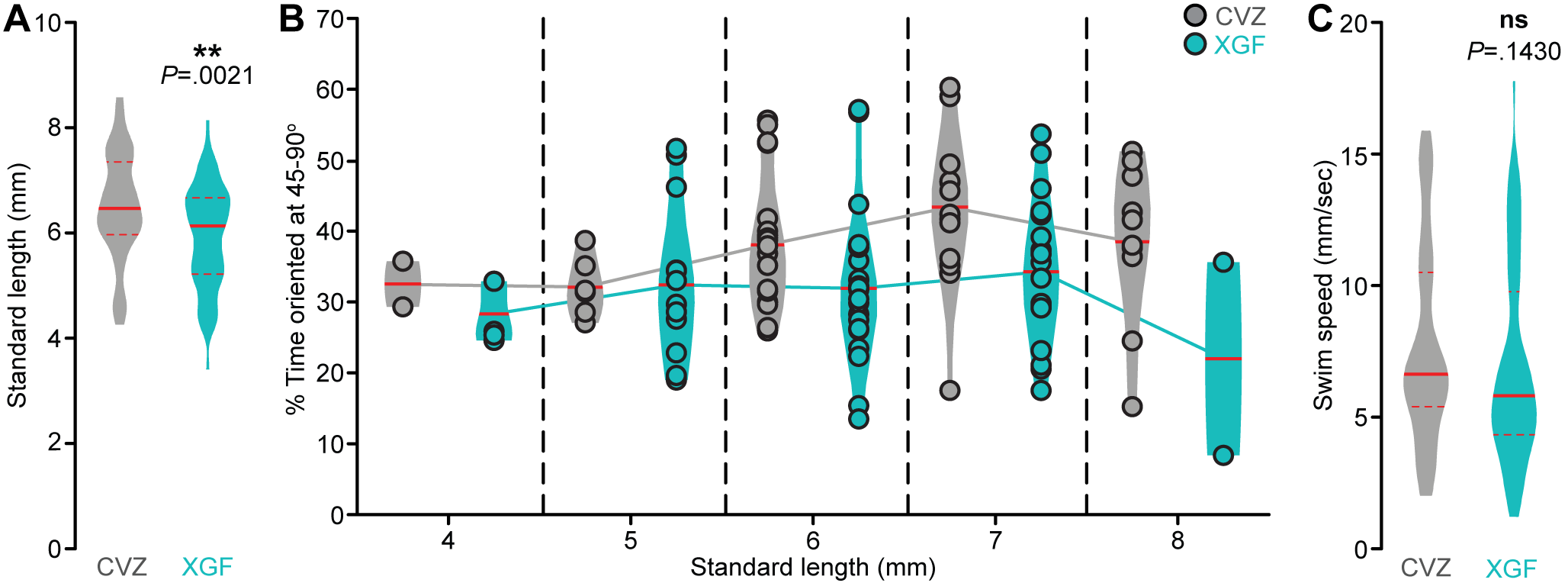

Supplement: S1 Fig — (A) Standard length is reduced in 14 dpf XGF (aqua) larvae relative to CVZ (gray) siblings (n = 57 CVZ and 66 GF larvae; unpaired t test). (B) Percent time oriented at 45–90° in XGF larvae and CVZ siblings, binned according to standard length. (C) Swim speed is not significantly different between XGF larvae and CVZ siblings (n = 55 CVZ and 67 GF larvae; Mann–Whitney U test). ns, not significant; **, P < .01. Solid red line represents the median; dotted red lines represent the upper and lower quartiles. Data underlying this figure are available on figshare: https://figshare.com/projects/Bruckner_et_al_Data/136756. (TIF) [file pbio.3001838.s001.tif]

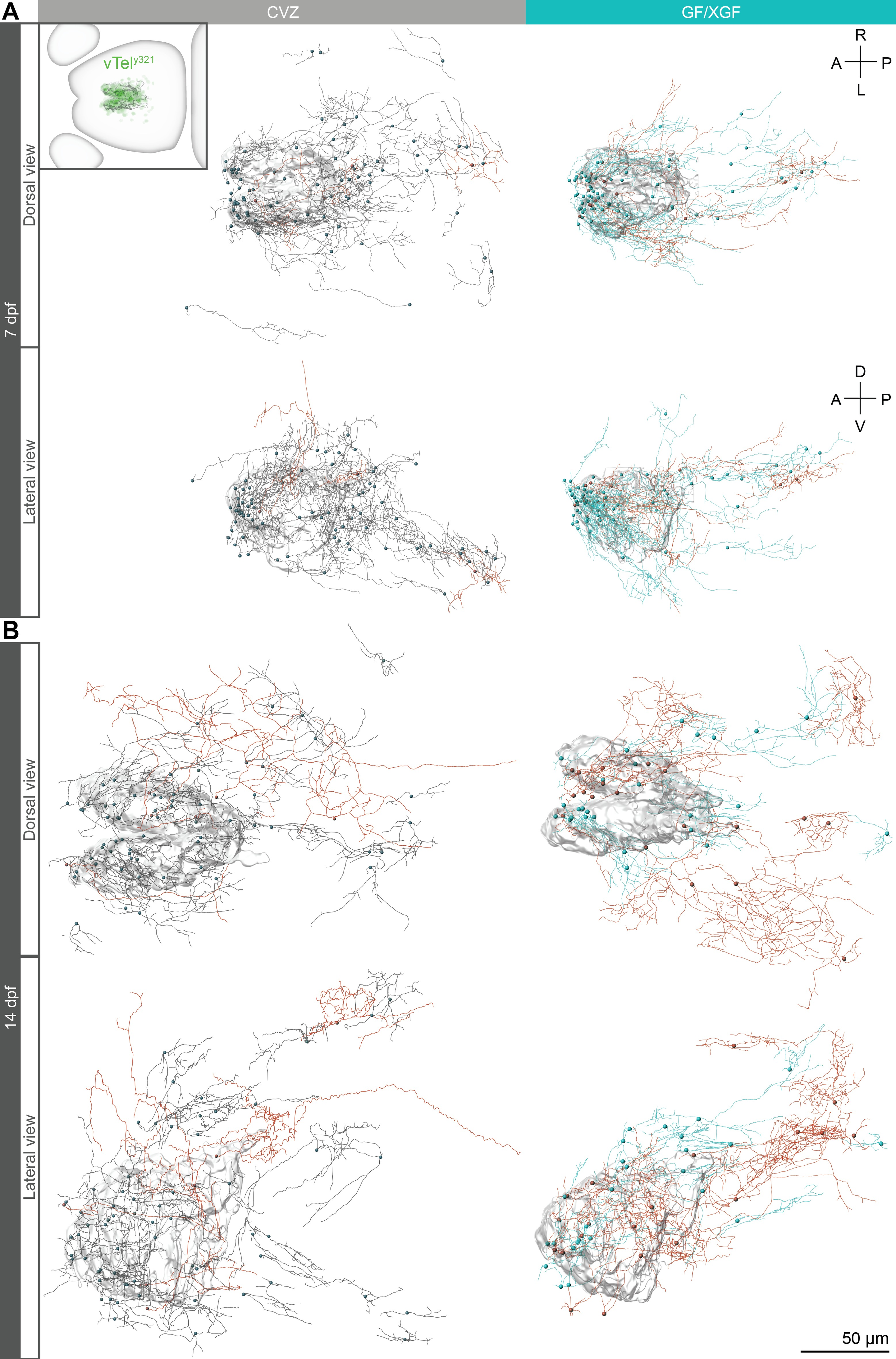

Supplement: S2 Fig — Dorsal (top) and lateral (bottom) views of vTely321 neurons from (A) 7 dpf CVZ (gray) and GF (aqua) larvae and (B) 14 dpf CVZ (gray) and XGF (aqua) larvae registered to an average vTely321 nucleus (transparent 3D model) from each condition and developmental stage. Average vTely321 nuclei do not incorporate sparse neuronal somata at the periphery, which are within the forebrain boundary. Neurons right of the dotted line in the factor analysis plots in Fig 3H and 3J are indicated in orange. (TIF) [file pbio.3001838.s002.tif]

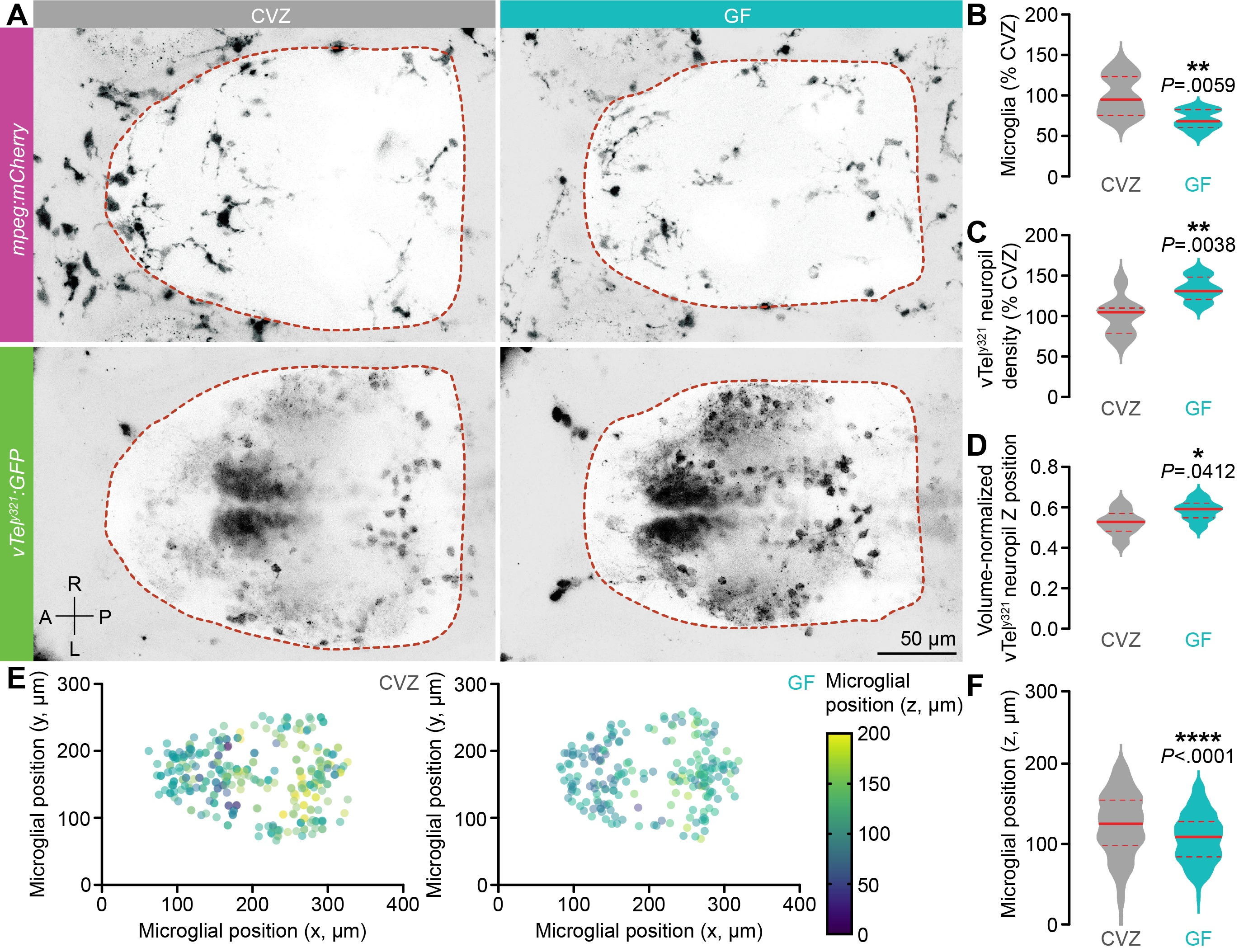

Supplement: S3 Fig — (A) Representative dorsal views of maximum-intensity projections of mpeg1:mCherryTg (microglia and macrophages, magenta) and vTely321 GFP (neurons, green) in 7 dpf CVZ (gray) or GF (aqua) larvae. Dotted lines indicate approximate forebrain boundary, segmented from the corresponding brightfield image. (B-D) The number of forebrain microglia, normalized to total forebrain volume (B), is reduced in GF larvae relative to CVZ siblings, while vTely321 neuropil density (C) and the position of the center of mass of the vTely321 neuropil, normalized to forebrain size (D), are increased in GF larvae relative to CVZ siblings (n = 8 CVZ and 8 GF larvae; unpaired t test). (E) 3D position of individual forebrain microglia from CVZ (left) and GF (right) larvae (n = 319 microglia from 8 CVZ larvae 208 microglia from 8 GF larvae). Z position is indicated by color. (F) Average microglial Z position is significantly reduced in GF larvae relative to CVZ siblings (n = 319 microglia from 8 CVZ larvae 208 microglia from 8 GF larvae; Welch’s t test). *, P < .05; **, P < .01; ****, P < .0001. Solid red line represents the median; dotted red lines represent the upper and lower quartiles. Data underlying this figure are available on figshare: https://figshare.com/projects/Bruckner_et_al_Data/136756 (TIF) [file pbio.3001838.s003.tif]

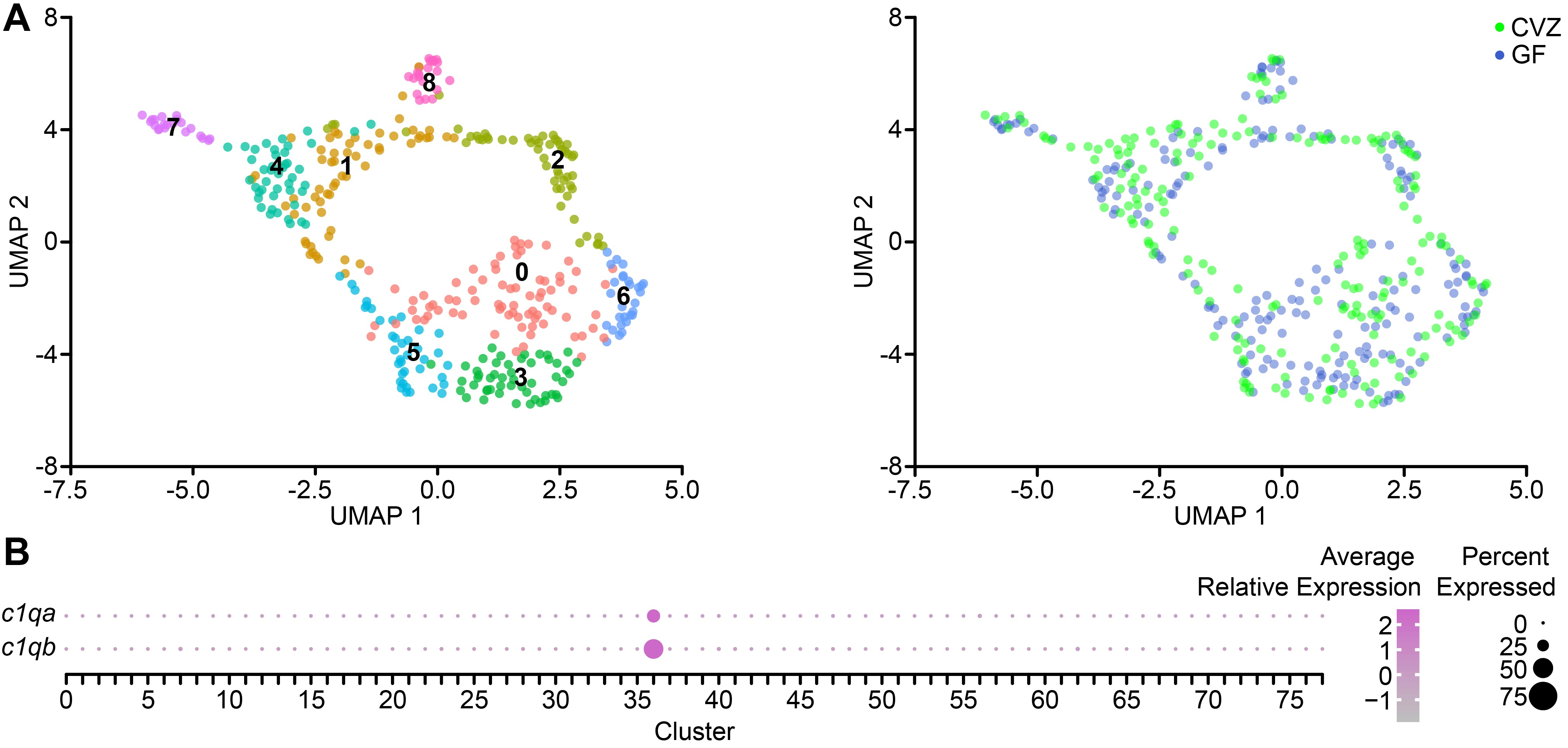

Supplement: S4 Fig — (A) The distribution of cells within each mpeg1.1+ Cluster 36 subcluster (left) is similar for cells from larvae raised CVZ (right, green) or GF (right, blue). (B) c1qa and c1qb expression is largely exclusive to Cluster 36 immune cells. Data underlying Fig 6A were also used to create (A) above, and with the data underlying (B), are available on figshare: https://figshare.com/projects/Bruckner_et_al_Data/136756. (TIF) [file pbio.3001838.s004.tif]
