# Supplementary material for: The microbiota promotes social behavior by modulating microglial remodeling of forebrain neurons
Source: PLoS Biol. 2022 Nov 1;20(11):e3001838. doi: 10.1371/journal.pbio.3001838 (PMC9624426; doi:10.1371/journal.pbio.3001838)
Supplement: Attachment — Submitted filename: BrucknerResponseToReviewers.docx [file pbio.3001838.s018.docx]

We thank the reviewers for considering our manuscript, and for the thoughtful suggestions they have provided for improvement. The reviewers were enthusiastic about our study, stating that it “…*is a fascinating study adding further evidence for the role of the microbiome in social behaviour*,” “…*is a very interesting paper, because it provides a link between the microbiota, social behavior, and microglia-neuron interactions*,” and “*the findings are of great interest*….”  We have made significant changes to the manuscript that we believe address the reviewers’ concerns, and that will hopefully clarify the results for our readers. Response to each individual critique is included below:

**Reviewer 1:**

*Bruckner et al.'s manuscript explores the mechanism by which the microbiota is able to modulate the development of behavioral traits, using the zebrafish as a model. They established a robust protocol allowing to raise gut bacteria-free embryos and larvae and showed in previous publications that microbiota is important for development of some* *specific innate behaviors. They also showed that a specific population of neurons in the ventral telencephalon was required for these behaviors. Here they test the hypothesis that the microbiota may be required for normal development of these telencephalic neurons.
They indeed observe that embryos raised without bacteria until 7dpf (GF/XGF) show abnormalities in these neurons. They show that the vTel population has increased neurite arborization compared to control. They also show that microglia in GF larvae is reduced in number in the telencephalon. They therefore test the reasonable hypothesis that lack of microbiota reduces forebrain microglia population, in turn leading to increased vTel neurite arborization. The findings are of great interest but the manuscript lacks clarity in places and requires more experiments to strengthen the conclusions made. The three main weaknesses are: i) the absence of evidence for telencephalic specificity in increase of neurite arborization and decreased microglia ii) the reduced amount of data directly supporting causality from low number of microglia to high amount of neurite branching, and iii) the very preliminary functional data suggesting c1q complement involvement in the reduced interaction
between neurons and microglia.*

*Specific concerns:*

*Tracing of the vTel in Figure S2 strongly suggest that the nucleus (and maybe the whole telencephalon) is smaller in the GF animals at both 7 and 14dpf. The authors need to measure total telencephalic volume to assess whether the forebrain is smaller in GF (only overall larval size was measured) and if so, whether the size difference is also present in other brain areas.*

Measurements of vTel^y321^ neurites and microglial abundance were normalized to total forebrain volume, which is the same in GF and CVZ larvae. These normalizations are indicated in the corresponding figure legends, for example for Figure 4: “(C) The number of forebrain microglia, normalized to total forebrain volume, is decreased in GF larvae relative to CVZ larvae.”

We also considered the possibility that the microbiota influences the number of cells in the vTel^y321^ nucleus. We found that the number of vTel ^y321^ neurons was slightly decreased in 7 dpf GF animals, but not in 14 dpf XGF animals, the age at which we tested social behavior. Estimates of vTel^y321^ nucleus size are included in Figure 2B and described on pg. 7: “The vTel^y321^ nucleus comprises an average of 229 neurons in 7 dpf CVZ larvae, which is slightly but significantly reduced in GF larvae (Fig 2A, B). However, 14 dpf XGF larvae that cannot execute normal social behavior have a similar number of vTel^y321^ neurons as their CVZ siblings, indicating that the microbiota does not influence *y321Et* promoter expression or modulate social behavior by promoting vTel^y321^ neuron proliferation (Fig 2A, B).”

*The increased arborization observed in vTel is compelling and extremely interesting. Is increased branching also observed in other neuronal populations? Or is it specific to vTel? The same question needs to be assessed for microglia. Is drop in microglia number telencephalon-specific or seen throughout the brain (author can normalize to tectal area to test forebrain specificity as tectal microglia is easy to image and quantify)?*

Whether the same mechanisms of microbial modulation of neuro-immune development that we observe in the forebrain also occur in other brain regions is an interesting question. Previous studies in rodents show that absence of the microbiota can have quite distinct influences in different brain regions (Nagpal and Cryan, 2021). Therefore, we focused on how the microbiota influences vTel, a brain region we previously showed is required for normal social behavior. We have added discussion of research describing microbiota influences across various brain regions on pg. 22: “How the microbiota influences neurodevelopment outside of the subpallium bears investigation in future studies. Based on the diverse neuronal and microglial phenotypes that have been reported in germ-free rodents [4,49,71–75], it is reasonable to expect that the microbiota modulates a variety of different neurodevelopmental mechanisms in brain regions other than the subpallium.”

*In the GF (Fig. 4A,B) and microglia morphants (Fig. 4E), the number of vTel neurons looks increased on the panels shown. Quantification of neuron numbers need to be provided for these two experiments in addition to neuropil density.*

We appreciate this point. Quantification of the number of vTel^y321^ neurons in GF and CVZ animals is included in Fig. 2B and described on pg. 7: “The vTel^y321^ nucleus comprises an average of 229 neurons in 7 dpf CVZ larvae, which is slightly but significantly reduced in GF larvae (Fig 2A, B). However, 14 dpf XGF larvae that cannot execute normal social behavior have essentially the same number of vTel^y321^ neurons as their CVZ siblings, indicating that the microbiota does not influence *y321Et* promoter expression or modulate social behavior by promoting vTel^y321^ neuron proliferation (Fig 2A, B).”

We quantified the number of vTel^y321^ neurons in *irf8* morphants relative to controls and found that the number of vTel^y321^ neurons is similar in both conditions. These results have been added to the manuscript in Fig. 4H and are described on pg. 13: “*irf8* morphants have essentially the same number of vTel^y321^ neurons as uninjected or random-control-injected animals (Fig 4H), suggesting that microglia are not significantly involved in forebrain neuronal apoptosis at 7 dpf.”

These results are also discussed on pg. 23: “The number of vTel^y321^ neurons is essentially the same in GF and CVZ fish, microglial reduction does not affect the number of vTel^y321^ neurons, and apoptotic neurons are largely absent by 6 dpf [56], so it is unlikely that the microbiota draws microglia to the developing forebrain by promoting apoptosis.”

*Differentiating microglia are present in the midbrain and forebrain as early as 3dpf. To further strengthen the causal link from less microglia to increased neurite branching, the authors should FACS GFP+ microglia from 4dpf and 5dpf control and inject them into GF larval forebrain at the same stage and assess whether injection rescues GF, reducing vTel branching and larval behavior.*

Whether FACS-sorted microglia from CVZ controls could rescue GF neuronal and behavioral phenotypes is an interesting question. Unfortunately, to our knowledge such an experiment has never been attempted before in zebrafish and is therefore not technically feasible within the timeline provided. Microglia infiltrate the zebrafish brain in two waves at 2.5 dpf and 14 dpf, so it is unlikely that FACS-sorted cells injected at 4 or 5 dpf will engraft and adopt their normal developmental functions. We discuss zebrafish microglial infiltration on pg. 4: “Microglia infiltrate the brain in multiple waves [25,26]. In zebrafish, this process begins with primitive microglia that differentiate in the rostral blood island, infiltrate the brain around 2.5 dpf, and persist through larval stages [27,28]. These microglia are eventually replaced by a second population that differentiates from hematopoietic stem cells in the dorsal aorta and infiltrates the brain beginning at approximately 14 dpf [26,29].”

It is also difficult to maintain zebrafish larvae in a GF state in any injection paradigm, so it would be difficult to exclude the effect of contaminating microbes when injecting FACS-sorted microglia into GF larvae. Though other mechanisms also likely influence vTel^y321^ neurite branching, the increase in vTel^y321^ neurite density following morpholino-mediated microglial reduction indicates a causal link between microglial abundance and vTel^y321^ neurite complexity as has been observed in other circuits and models.

*The scRNAseq analysis in Fig 6 is done from clustering RNAseq from whole larvae. It is therefore not surprising to see subtle difference in the signature of gene expression in the microglia clusters as some of these cells would be localized in and/or around the gut and experiencing a very different surveying landscape. Not sure whether the proteosomal and lysosomal difference identified is meaningful for the telencephalic microglia specifically. Unless the authors can better articulate the meaning of the difference in GO terms for the telencephalon population specifically and validate it, I would remove this part of the manuscript.*

We used a 75-gene core microglial fingerprint to separate microglia from other *mpeg1.1*^+^ cells, including intestinal macrophages, which is included in Fig 6 and described on pg. 16: “The common developmental origin and extreme transcriptional similarity between microglia and macrophages decreases the utility of individual “marker” genes, even when used in multi-gene combinations [19]. To address this issue, we mapped expression of a 75-gene core microglial fingerprint to our nine clusters (Fig 6B, Supplemental Table 1). Critically, though individual genes in this list may be expressed in both macrophages and microglia, combinatorial expression of the entire fingerprint is unique to microglia. A significantly greater percentage of cells (for many genes > 80%) in subclusters 1, 2, and 4 express the microglial fingerprint at higher levels than the other clusters, indicating that the cells in these clusters are predominantly microglia.”

Previous research suggests that the three microglial subsets we identify among *mpeg1.1*^+^ cells do not overlap with tissue-resident macrophages outside of the brain (Mazzolini et al., 2020; Wu et al., 2020). Therefore, our analysis of the influence of the microbiota on gene expression in these cells does not include changes in and/or around the gut and the changes in gene expression presented in the GO analysis portion of Fig 6 are specific to CNS microglia. As indicated on pg. 16, it is unclear whether intestinal macrophages are included among the other *mpeg1.1*^+^ clusters: “our searches for known markers of macrophage subsets, including border associated, M1, and M2 macrophages, did not reveal unique identities among the macrophage clusters [63–65]. It is possible that these macrophage clusters represent biologically relevant cell types or states, but the lack of pro-inflammatory challenge in this dataset likely minimizes the representation of activated macrophages and small sets of qualitative markers may be insufficient to distinguish macrophage subtypes.”

On pg. 24, we include discussion of previous research suggesting that the ramified microglia we identify (cluster 4) are enriched in synapse-rich regions like the ventral forebrain and preoptic commissure: “The three microglial clusters we identify likely include amoeboid, ramified, and proliferative microglia, and represent previously-identified microglial heterogeneity across the zebrafish brain [64,66]. Our *ccl34b.1*^+^ population ("amoeboid" cluster 1) likely corresponds to optic tectum-enriched neurogenic associated microglia (NAMs) and our *ccl34b.1*^-^ population (“ramified” cluster 4) likely corresponds to hindbrain-enriched synaptic-region associated microglia (SAMs) [64].”

*I am a bit worried about the fluorescent in situ shown on Fig 7. There is a substantial amount of signal outside of the cells in which the gene is expressed. How do the authors recognize signal vs background?*

We thank the reviewer for pointing out this concern. We have added detail explaining how *c1qa* fluorescent in situ images were segmented to specifically quantify signal within microglia to the Materials and Methods on pg. 34: “*c1qa* RNA localization was quantified by semi-automatic signal-based segmentation using the surfaces function in Imaris. Briefly, *mpeg1:mCherry* signal was used to mask each image, create a 3D region of interest for analysis of *c1qa* signal in each cell, and estimate microglial volume. Imaris Spots was used to detect and quantify *c1qa* puncta in each cell. Intensity-based thresholds were applied equally across all images and conditions.” Additionally, we explain the negative and positive control probes that were used to assess signal specificity on pg. 32: “A custom probe against *gad1b* was used as positive control, and the ACD RNAscope 4-plex Negative Control Probe was used as negative control (ACD #321831), and we observed no difference in control probe signal between conditions.”

*The c1q MO experiment is affecting c1q from the start of its expression everywhere it is expressed. The fact that there is an increase in neuropil in the telencephalon of the MO injected could be very indirect, for instance by affecting the gut of the embryo, which in turn would lead to telencephalic phenotype. If the authors want to address this, they should FACS mpeg:mCherry cells from c1q crispants (MO embryos do not deal with FACS well), inject them into forebrain of normal 3dpf vTelGFP larvae and image neuropil at 7dpf.*

The reviewers are correct to point out that the *c1q* morpholinos affect *c1q* expression ubiquitously and constantly. However, our scRNAseq data included in Fig S4 and described on pg. 20 suggest that, at least at 6 dpf, *c1qa* and *c1qb* are not strongly expressed outside of mpeg1.1^+^ cluster 36: “Complement pathway genes, especially *c1q*, can also mediate synaptic remodeling when expressed and secreted by neurons and astrocytes [32], and our morpholino injection likely results in ubiquitous knockdown. However, *c1qa* and *c1qb* are almost exclusively expressed by the microglia-containing Cluster 36 (Fig S4B), so increased vTel^y321^ neurite density likely results from impaired microglial remodeling rather than misfunction in neurons, astrocytes, or tissues outside the CNS.” It is therefore unlikely that the effects of *c1q* knockdown that we observe in neurons are the result of effects on other *c1q*-expressing cell types. Similar to the point raised above, generating *c1q* crispants and FACS microglia are not technically feasible in the timeline provided and would dramatically expand the scope of this manuscript.

*Minor point: 
As there is not difference in microglia morphology or kinetics, Fig 5 should be moved to suppl. Data*

We believe that negative results also belong among the main figures when they are of significant interest to the broader field and critical to the manuscript narrative. The results presented in Fig 5 represent an important contrast with previous results in murine model systems, and this is discussed on pg. 23-24: “Microglia in GF mice are also metabolically dysfunctional, larger, less mature, less responsive to LPS challenge, and more abundant in the cortex, corpus callosum, hippocampus, olfactory bulb, and cerebellum than in specific pathogen free (SPF) controls [49,74,75]. Contrary to these murine phenotypes, zebrafish forebrain microglia are reduced in GF larvae relative to CVZ controls and forebrain microglial morphology does not appear influenced by the microbiota. We also do not observe an effect on the ratio of amoeboid and ramified microglia. How can these results be reconciled? One possibility is that the microbiota has differential effects on microglia across the brain, as microglial subtypes are heterogeneously distributed [64]. Microglia are drawn into the brain by chemokine signaling and neuronal apoptosis before 4 dpf, prior to microbiota colonization [28,56,88]. The number of vTel^y321^ neurons is essentially the same in GF and CVZ fish, microglial reduction does not affect the number of vTel^y321^ neurons, and apoptotic neurons are largely absent by 6 dpf [56], so it is unlikely that the microbiota draws microglia to the developing forebrain by promoting apoptosis. It is also possible that the microbiota has differential effects on early larval and juvenile microglia, which have distinct developmental origins and neuro-immune functions [25]. Further study will be necessary to gain a deeper understanding of the role of the microbiota in promoting or suppressing microglial development or dynamics and how this varies across brain regions, developmental time, and taxa.”

**Reviewer 2:**

*Bruckner et al. report interesting evidence that the microbiota controls social behavior in zebrafish by influencing the localization and function of microglia, which in turn regulate the arbors of forebrain neurons that modulate social behavior.

Key findings include that social behavior at 14 dpf is altered in animals raised in germ-free conditions for 0-7 dpf, and that arbors of forebrain neurons controlling this behavior are more dense in these animals.  Microglia are somewhat less abundant in the forebrain of GF animals.  The abnormalities in the forebrain of GF animals can be at least partially rescued by diverse, individual bacterial taxa.   Morphants with reduced microglia have increased forebrain neuropil, apparently providing the first evidence that microglia prune neuronal arbors in zebrafish.  Analysis of scRNA-seq data led the authors to examine c1q expression and function; they obtain evidence that c1q expression is reduced in microglia from GF animals, and that c1q knockdown increases forebrain neuropil.

This is a very interesting paper, because it provides a link between the microbiota, social behavior, and microglia-neuron interactions.  Some of the effect sizes are small, but overall the study is convincing.  Below are some points that the authors should address, with additional experiments and/or revisions to the text.

What is the critical period for the effect of microbiota on social behavior?  i.e., does restoration of microbiota at 6 dpf, 5 dpf, … rescue subsequent social behavior?  The social behavior assays are useful at stages well past when the authors examined.  Do the social abnormalities in XGF animals persist to adulthood (or at least past 14 dpf)?*

These are interesting questions. Inoculation with a normal microbiota at 7 dpf does not restore normal neuronal or social phenotypes at 14 dpf, so the critical period for the effect of the microbiota on social behavior is likely between when the gut becomes patent at 4 dpf and when we inoculate with a normal microbiota at 7 dpf. As the reviewer suggests, it would be interesting to know whether inoculation before 7 dpf is sufficient to restore social behavior in GF larvae, and this question will be explored in future work. This is discussed on pg. 20-21: “We observed that the microbiota is required early for normal social behavior exhibited at least a week later, suggesting that the microbiota influences social behavior by modulating neurodevelopment during an early sensitive period. Zebrafish social phenotypes significantly increase in complexity from 10-16 dpf [5]. Our observations suggest that the neuronal circuitry that facilitates this behavior requires microbial input as it is being built, likely between when the gut becomes patent at 4 dpf and when we assayed neuro-immune phenotypes and colonized with a normal microbiota at 7 dpf.”

Though it may be of interest to see how long the cellular phenotypes we observe persist after colonization at 7 dpf, the deficit in vTel^y321^ neurite complexity is quite stable over half the life span of 14 dpf larvae in the presence of a normal microbiota. We would therefore not expect neuronal or social phenotypes to recover with further development; however, these experiments are beyond the scope of this manuscript. The effect of the microbiota on adult phenotypes has been explored in other models, so in this manuscript we specifically addressed how the microbiota influences early social neurodevelopment, as discussed on pg. 21: “Previous work in other organisms has largely focused on how the microbiota influences adult neurodevelopmental phenotypes, therefore we decided to take advantage of the strengths of zebrafish as a model to understand how the microbiota influences neurodevelopment as the forebrain circuitry required to perform social behavior is being built.”

*It would strengthen the case for causality if the authors can link the timing of changes in microglia and neuronal arbors to the timing of social deficits discussed above.  In other words, does the microbiota critical period for social behavior coincide with changes in the arbors or localization of microglia?*

Our results included in Fig 1, Fig 2, and Fig 3 indicate that at 7 dpf, in the critical window when the microbiota is required for development of typical social behavior that occurs a week later, the microbiota influences vTel^y321^ neuronal arborization and forebrain microglial abundance. We also provide evidence that vTel^y321^ neuronal arbors normally continue to elaborate between 7 dpf and 14 dpf, when social phenotypes begin to be expressed (Stednitz and Washbourne, 2020), and that the presence of a normal microbiota during this time period is insufficient to restore typical neuro-immune phenotypes and social behavior. We conclude that continued vTel^y321^ arborization between 7-14 dpf is important for normal social behavior expression at 14 dpf. We have added text to the discussion on pg. 21 emphasizing this point: “Zebrafish social phenotypes significantly increase in complexity from 10-16 dpf [5]. Our observations suggest that the neuronal circuitry that facilitates this behavior requires microbial input as it is being built, likely between when the gut becomes patent at 4 dpf and when we assayed neuro-immune phenotypes and colonized with a normal microbiota at 7 dpf.”

*Another other experiment that would strengthen the microbiota-microglia-arbor linkage proposed by the authors would be to examine social behavior in irf8 mutants (or other mutants lacking microglia).  Similarly, are microglia required for mono-associated microbes to rescue neuropil density (i.e. repeat experiment in Figure 4D in mutants lacking microglia)?*

We agree that examining social behavior in zebrafish lacking microglia strengthens the

linkage between the microbiota, microglia, and vTel^y321^ neuronal arbors. We have added a comparison of social phenotypes at 14 dpf in *irf8* morphants and random-control-injected siblings to Figure 4, and these results are described on pg. 13-14: “To address whether microglial remodeling of vTel^y321^ arborization is required for normal social behavior, we assayed social behavior phenotypes as described above in 14 dpf larvae in which microglia have been reduced with morpholino against *irf8*. Compared to random-control-injected siblings, *irf8* morphants spent significantly less time in close proximity to the stimulus fish (Fig 4I). Though *irf8* morphants also appeared to spend slightly less time than random-control-injected siblings oriented at 45-90^o^ relative to the stimulus fish, this difference was not statistically significant (Fig 4J). As for XGF fish, impaired social behavior in *irf8* morphants cannot be explained by impaired larval motility (% time in motion; random control, 39.92 ± 2.07, *n* = 39 larvae; *irf8* MO, 36.32 ± 2.22, *n* = 34 larvae; *P* = .2391, Unpaired t-test).” These results suggest a more subtle phenotype than we observe in XGF larvae, as social place preference is significantly impaired but social orienting is trending towards impaired. This difference is consistent with the fact that *irf8* knockdown exclusively impairs microglial abundance while GF fish exhibit both impaired microglial abundance and impaired microglial function.

*When is the reduction in forebrain microglia first apparent in GF animals?  Are microglia also reduced in other areas of the CNS?*

The reviewer poses interesting questions. As described above, we sought to focus our assessment of neuro-immuno phenotypes in GF larvae on 7-14 dpf, as we hypothesized that social behavior circuitry that includes vTel^y321^ neurons would be established during or just before the time at which we have observed the behavioral repertoire expanding to include social behavior (Stednitz and Washbourne, 2020). On pg. 23, we discuss previous studies that have observed increased microglial abundance in several brain regions in GF mice: “murine microbiota disruption impairs responses to social novelty and alters microglial morphology and reactivity in the hippocampus and cortex [77,87]. Microglia in GF mice are also metabolically dysfunctional, larger, less mature, less responsive to LPS challenge, and more abundant in the cortex, corpus callosum, hippocampus, olfactory bulb, and cerebellum than in specific pathogen free (SPF) controls [49,74,75]. Contrary to these murine phenotypes, zebrafish forebrain microglia are reduced in GF larvae relative to CVZ controls and forebrain microglial morphology does not appear influenced by the microbiota.” Rather than attempting to identify the homologous zebrafish brain regions and replicate these results, we focused our analysis on how the microbiota influences forebrain-resident microglia specifically during early development.

*Relative to the original irf8 morpholino paper (Li et al. 2011), the knockdown reported by the authors seems very week.  Why do so many microglia (apparently more than half) remain in the irf8 morphants?*

The reviewer makes an excellent point. We now address these questions in the results on pg. 13: “Notably, the reduction in microglia that we observe in 7 dpf *irf8* morphants is not as dramatic as reported at 3 dpf, potentially due to waning morpholino efficacy or local microglial proliferation [57,58].” Despite this, the microglial reduction we observe in 7 dpf *irf8* morphants is comparable to that observed in GF larvae vTel^y321^ neuronal arbors and increases vTel^y321^ neurite density relative to uninjected or random-control injected siblings.

*The c1q morphant experiment is not as convincing as the other experiments reported in the paper.  What is the knockdown efficiency?  The increase in neuropil density seems small.  Is it possible to perform a rescue experiment in which c1q is restored to the morphants? Are there social deficits in c1q morphants?  Analysis of a c1q mutant would greatly strengthen the conclusions of this section of the paper.*

As with *irf8* morphants above, we strengthened the link between microbiota, microglial *c1q* function, and vTel^y321^ neuronal arbors by measuring social behavior in *c1q* morphants, added these results to Figure 7, and described them on pg. 19: “To address whether *c1q* abrogation alone is sufficient to induce social behavior deficits, we assayed social behavior phenotypes in *c1q* morphants at 14 dpf. Unlike *irf8* knockdown, *c1q* knockdown does not significantly affect the amount of time that larvae spend in close proximity to (Fig 7H) or oriented at 45-90^o^ relative to (Fig 7I) the stimulus fish. Larval motility is also unaffected by *c1q* knockdown (% time in motion; random control, 41.08 ± 2.14, *n* = 32 larvae; *c1q* MO, 38.19 ± 3.01, *n* = 31 larvae; *P* = .4351, Unpaired t-test).”

As the reviewer identifies, it would be interesting to compare *c1q* knockdown efficiency with the reduction in *c1q* expression that we observe in GF larvae. Similar to our experiments reducing forebrain microglia, we expected that *c1q* knockdown alone would not impair vTel^y321^ neurite density to the same extent as when both microglial abundance and function are impaired in GF larvae. We now include comparison of the neurite density phenotypes in *irf8* morphants, *c1q* morphants, and *GF* larvae on pg. 19-20: “*c1q* knockdown increases vTel^y321^ neurite density by 21% (Cohen’s *d*: 0.86), significantly less than the 26% increase observed following *irf8* knockdown (Cohen’s *d*: 0.93) or the 33% increase (Cohen’s *d*: 1.73) in GF larvae relative to CVZ controls. Though we cannot rule out differences in recovery of gene expression following morpholino injection, these results suggest that microbial modulation of both microglial abundance and microglial function influence social behavior via convergent effects on vTel^y321^ neuronal arborization. It is possible that *c1q* knockdown does not impair social behavior because the specific disruption of arbor pruning in *c1q* morphants is not dramatic enough to impair circuit function, or that other circuit components are able to compensate for the relatively mild impairment of vTel^y321^ neurite density.”

**Reviewer 3:**

*This is a fascinating study adding further evidence for the role of the microbiome in social behaviour

1. Which microbiota was used for conventionalizing? Was there a difference between CV and CVZ controls?*

These are excellent questions. Conventionalization of GF larvae is described in the materials and methods on pg. 29: “CVZ flasks were inoculated with 200 µl water from the parental tank immediately following the GF derivation procedure.”

Assessing whether there is a difference between CV and CVZ controls in our experiments could be a useful tool, in future studies, for studying how variable microbiota composition contributes to neurodevelopment. In this work, we used the comparison between GF and CVZ larvae to control for any influence of our gnotobiotic processes on the phenotypes observed, as is the standard for gnotobiology best practices in zebrafish (Melancon et al., 2017).

*2. Was hyperactivity observed in GF larvae as has been reported previously (eg Phelps et al 2017) ?*

It is certainly interesting that other groups have observed hyperactivity in GF larvae at 10 dpf. As described in Fig 1 and Fig S1, we did not observe an effect of the microbiota on motility or swim speed at 7 dpf or 14 dpf, respectively. Phelps et al. also did not observe a difference at 7 dpf, and did not assay later stages. Therefore, our results may not actually differ from theirs. These considerations are discussed on pg. 21: “The zebrafish microbiota is also required for normal activity and anxiety-like behavior, suggesting that these phenotypes could be linked to the impaired social behavior we observe [45,69]. However, GF larvae and their CVZ siblings perform similarly in our optomotor assay and motor behavior remains unaffected in 14 dpf XGF larvae, thus the social defects we observe in XGF larvae may instead result from impaired integrative circuits downstream of sensory input and upstream of motor output.”

*3. Was the body length (and hence development state) normalized for arborization and subsequent experiments?*

This is an excellent question. As described in response to Reviewer 1 above, measurements of neurite density and microglial abundance include normalization to total forebrain volume, which is similar in CVZ and GF larvae. Therefore, our results suggest that delayed developmental state cannot solely account for the neuro-immune effects of the microbiota that we describe.

*4. Was the microglia distribution evaluated at 14 dpf XGF and CVZ as was done for arbor analysis?*

Whether microglial populations remain affected later in development following colonization is an interesting question, though tangential to the narrative of our inquiry. As indicated above, we have focused our analysis on how the microbiota influences neurodevelopment via microglia specifically during early development. Our results indicate that microbial colonization at 7 dpf is unable to restore neuronal arborization or social behavior at 14 dpf, so any effect of the microbiota on microglial abundance during that time is clearly insufficient to influence circuit function.

*5. Microglial abundance and neuropil density was ascertained at what age in experiments with morpholino against the microglial gene irf8?*

We thank the reviewer for identifying this oversight, which is now corrected on pg. 13: “we reduced forebrain microglia by injecting embryos with a previously validated morpholino against the microglial gene *irf8* [57] and measured microglial abundance and vTel^y321^ neuropil density at 7 dpf as described above (Fig 4E).”

*6. Was social behaviour assayed upon administration of different bacterial strains?*

Whether individual bacterial strains that restore neuronal arborization and microglial abundance also rescue social behavior is an interesting question. However, interpretation of this experiment would be complicated by the fact that, for technical reasons, monoassociation cannot be maintained once the larvae are fed at 7 dpf, colonized with a normal microbiota, and raised until social behavior is apparent at 14 dpf.

*Minor
The term dysbiosis is heavily disputed in the field and should be avoided*

We appreciate this suggestion and have replaced the term dysbiosis throughout the manuscript.

*The authors articulate and justify very well the concept of using zebrafish as a model of social behaviour but could also point out how there is increasing emphasis on its role as an excellent tool to dissect mechanisms in microbiota-gut-brain axis function (eg PMID: 34653349)*

We appreciate this suggestion and have addressed this point, with the additional reference, on pg. 3: “Zebrafish are increasingly employed to explore the microbiota-gut-brain axis and are an excellent model for understanding how the microbiota influences development of the ‘social brain’ and generating insights to inform interventions in humans [4].”

**References**

Mazzolini, J., Le Clerc, S., Morisse, G., Coulonges, C., Kuil, L.E., Ham, T.J., Zagury, J., and Sieger, D. (2020). Gene expression profiling reveals a conserved microglia signature in larval zebrafish. Glia *68*, 298–315.

Melancon, E., Gomez De La Torre Canny, S., Sichel, S., Kelly, M., Wiles, T.J., Rawls, J.F., Eisen, J.S., and Guillemin, K. (2017). Best practices for germ-free derivation and gnotobiotic zebrafish husbandry. In The Zebrafish - Disease Models and Chemical Screens, (Elsevier), pp. 61–100.

Nagpal, J., and Cryan, J.F. (2021). Microbiota-brain interactions: Moving toward mechanisms in model organisms. Neuron.

Stednitz, S.J., and Washbourne, P. (2020). Rapid Progressive Social Development of Zebrafish. Zebrafish.

Wu, S., Nguyen, L.T.M., Pan, H., Hassan, S., Dai, Y., Xu, J., and Wen, Z. (2020). Two phenotypically and functionally distinct microglial populations in adult zebrafish. Sci. Adv. *6*, eabd1160.
